# Prodrug-Activating Chain Exchange (PACE) converts targeted prodrug derivatives to functional bi- or multispecific antibodies

**DOI:** 10.1515/hsz-2021-0401

**Published:** 2022-04-26

**Authors:** Steffen Dickopf, Can Buldun, Vedran Vasic, Guy Georges, Carina Hage, Klaus Mayer, Matthias Forster, Uwe Wessels, Kay-Gunnar Stubenrauch, Jörg Benz, Andreas Ehler, Matthias E. Lauer, Philippe Ringler, Sebastian Kobold, Stefan Endres, Christian Klein, Ulrich Brinkmann

**Affiliations:** Large Molecule Research (LMR), Roche Innovation Center Munich, Roche Pharma Research and Early Development (pRED), Penzberg, Germany; Discovery Oncology, Roche Innovation Center Munich, Roche Pharma Research and Early Development (pRED), Penzberg, Germany; Pharmaceutical Sciences (PS), Roche Innovation Center Munich, Roche Pharma Research and Early Development (pRED), Penzberg, Germany; Small Molecule Research, Roche Innovation Center Basel, Roche Pharma Research and Early Development (pRED), Basel, Switzerland; Chemical Biology, Roche Innovation Center Basel, Roche Pharma Research and Early Development (pRED), Basel, Switzerland; Center for Cellular Imaging and Nano Analytics, Biozentrum University of Basel, Basel, Switzerland; Center of Integrated Protein Science Munich (CIPS-M) and Division of Clinical Pharmacology, Department of Medicine IV, University Hospital, Ludwig Maximilians University of Munich, German Center for Lung Research (DZL), Munich, Germany; German Center for Translational Cancer Research (DKTK), Partner Site Munich, Munich, Germany; Discovery Oncology, Roche Innovation Center Zurich, Roche Pharma Research and Early Development (pRED), Schlieren, Switzerland

**Keywords:** bsAb, immunotherapy, prodrug, T cell engager

## Abstract

Driven by the potential to broaden the target space of conventional monospecific antibodies, the field of multi-specific antibody derivatives is growing rapidly. The production and screening of these artificial proteins entails a high combinatorial complexity. Antibody-domain exchange was previously shown to be a versatile strategy to produce bispecific antibodies in a robust and efficient manner. Here, we show that the domain exchange reaction to generate hybrid antibodies also functions under physiological conditions. Accordingly, we modified the exchange partners for use in therapeutic applications, in which two inactive prodrugs convert into a product with additional functionalities. We exemplarily show the feasibility for generating active T cell bispecific antibodies from two inactive prodrugs, which per se do not activate T cells alone. The two complementary prodrugs harbor antigen-targeting Fabs and non-functional anti-CD3 Fvs fused to IgG-CH3 domains engineered to drive chain-exchange reactions between them. Importantly, Prodrug-Activating Chain Exchange (PACE) could be an attractive option to conditionally activate therapeutics at the target site. Several examples are provided that demonstrate the efficacy of PACE as a new principle of cancer immunotherapy *in vitro* and in a human xenograft model.

## Introduction

The therapeutic applicability of numerous antibody derivatives in clinical development is limited by unfavorable properties such as safety or pharmacokinetics (Kamath 2016; Vafa and Trinklein 2020; Walker 2004). In some instances, such problems can be directly linked to their targets or mode of action. A prominent example for an antibody-based principle whose applications are currently limited by the availability of suitable tumor specific targets (particularly for solid tumors) are T cell recruiting bispecific antibodies (TCBs), which crosslink polyclonal T lymphocytes and tumor cells to induce potent T cell-mediated cytotoxicity at the tumor site (Ellerman 2019; Vafa and Trinklein 2020).

The success of blinatumomab in acute lymphoblastic leukemia (ALL) after its market launch in 2014 led to rising attention in the cancer immunotherapy field and the initiation of numerous (pre-) clinical trials for TCBs targeting different tumor-associated antigens (TAA) in solid or hematopoietic indications (Kobold et al. 2018; Labrijn et al. 2019; Strohl and Naso 2019; Topp et al. 2014). Blinatumomab is a bispecific antibody in the tandem single chain Fv (tandem scFv) – format that is capable of simultaneously targeting CD19 on B cells and the TCR subunit CD3ε. This transcellular binding induces the formation of an artificial immune synapse and potent T cell-mediated killing of CD19-positive B cells (Dickopf et al. 2020; Hoffman and Gore 2014; Klinger et al. 2012). Due to its small size and the lack of an Fc region, blinatumomab is rapidly cleared from the system and hence has to be applied by continuous infusion.

With growing interest in T cell engager molecules and many preclinical evaluations of this mode of action, it became apparent that the selection of tumor-specific antigens represented a major challenge. Even the targeting of antigens with relatively low expression in healthy tissue often leads to an undesired activation of T cells, causing on-target off-tumor toxicity (Ellerman 2019; Vafa and Trinklein 2020). A prominent example is the epidermal growth factor receptor (EGFR), which besides being overexpressed in colon cancer, is also present in non-malignant tissues including skin, liver and kidney. Several studies reported narrow therapeutic windows when targeting EGFR with TCBs (Boustany et al. 2018; Desnoyers et al. 2013; Lutterbuese et al. 2010), and many other clinically relevant targets are also unsuitable as targets for conventional TCBs due to safety concerns (Dickopf et al. 2020; Ellerman 2019; Slaga et al. 2018).

One option to address such limitations is the design of antibody-prodrugs that are inactive in isolation but become activated at the site (cell or tissue) where efficacy is desired. Examples for antibody prodrug approaches include protease-activatable antibodies that contain a masking moiety that shields the ‘problematic’ binder. The masking moiety is connected via a protease-cleavable linker, which becomes cleaved by enzymes that are frequently expressed in the tumor microenvironment (Trang et al. 2019). Similar efforts by CytomX, Amunix and Maverick Therapeutics showed that conditional activation by proteolytic cleavage can extend TCB therapeutic windows for targets with reduced tumor specificity (Desnoyers et al. 2013; Panchal et al. 2020; Sim 2017). Boustany and colleagues showed that protease-activatable EGFR-targeted Probodies^®^ have a 60-fold higher tolerable dose in non-human primates while retaining an efficacy similar to the unmasked entity in mice (Boustany et al. 2018). This protease-activatable probody concept has subsequently been expanded by similar approaches to elicit reduced multi-organ immune toxicity of an anti-CTLA4 and 4-1BB antibody in mice (Etxeberria et al. 2021; Pai et al. 2019). We recently expanded this approach towards protease-activated folate receptor and mesothelin-targeting 2 + 1 TCBs using anti-idiotypic scFv masks (Geiger et al. 2020).

Although protease-activatable antibody prodrugs have made progress, their activation depends on the presence and specificity of tumor-associated proteases as well as the expression of the respective tumor antigen by target cells. A major challenge is the design of protease-activation sequences with exclusive specificity for tumor-specific proteases. Furthermore, it is critical that tumor specific proteases are exclusively localized on tumors and have no relevant activity in the circulation (Poreba 2020). In an alternative preclinical approach for conditional T cell activation Kamata-Sakurai and colleagues exploited extracellular adenosine triphosphate levels (exATP) in the tumor microenvironment and engineered ATP-dependent binders (Kamata-Sakurai et al. 2021; Keenan and Fong 2021). Benaszek et al. used two scFv antibodies against two different antigens, each fused to either an unpaired VH or VL of a CD3 binder, which they termed hemibodies. By simultaneous binding to a tumor cell, both antibodies align and restore into a T cell-engaging antibody (Banaszek et al. 2019).

Here, we present Prodrug-Activating Chain Exchange (PACE) as a new concept for generation of antibodies with prodrug functionality. PACE is based on a recent chain exchange technology (Format Chain Exchange [ForCE] [Dengl et al. 2020]) developed for robust high-throughput generation of bispecific antibody matrices *in vitro*. We adapted this technology to enable chain exchange not only under controlled chemical conditions *in vitro*, but also under physiological conditions. Accordingly, we placed the exchange-driving ForCE-CH3 domains into the bispecific TriFab format (Dickopf et al. 2019; Mayer et al. 2015). PACE does not rely on proteolytic cleavage for activation, but instead achieves activation by the simultaneous presence of two educts in close proximity. Thereby, two antibody derivatives that carry functional cell surface binders and additionally a nonfunctional (prodrug) binder become converted to a functional bi (or tri)-specific antibody. As opposed to hemibodies (Banaszek et al. 2019), this molecular architecture can be described as a tumor targeting Fab that is linked to a fully assembled inactive Fab-like V-CH3 fusion, with the goal to avoid unpaired VH and VL domains exposing hydrophobic interfaces, and thus to improve the biophysical properties of the molecule. Upon encountering each other (e.g., on/in tumors), the prodrugs exchange their heavy chains and thereby reconstitute active functional binders in TriFab formats. We demonstrate that PACE enables the generation of defined and well-behaved prodrugs, which become activated and therapeutically effective under physiological conditions in cell culture and in an animal model. Examples include PACE-prodrugs for inactivation and re-activation of hapten binders, as well as prodrugs that harbor an inactivated CD3-binder.

## Results

### Generation of bispecific antibodies under physiological conditions *in vitro*

The molecular mechanism that enables chain-exchange-based prodrug activation is an adaptation of our recently developed technology (ForCE) for robust, high-throughput *in vitro* generation of bispecific antibodies (Dengl et al. 2020).

**Figure 1:**
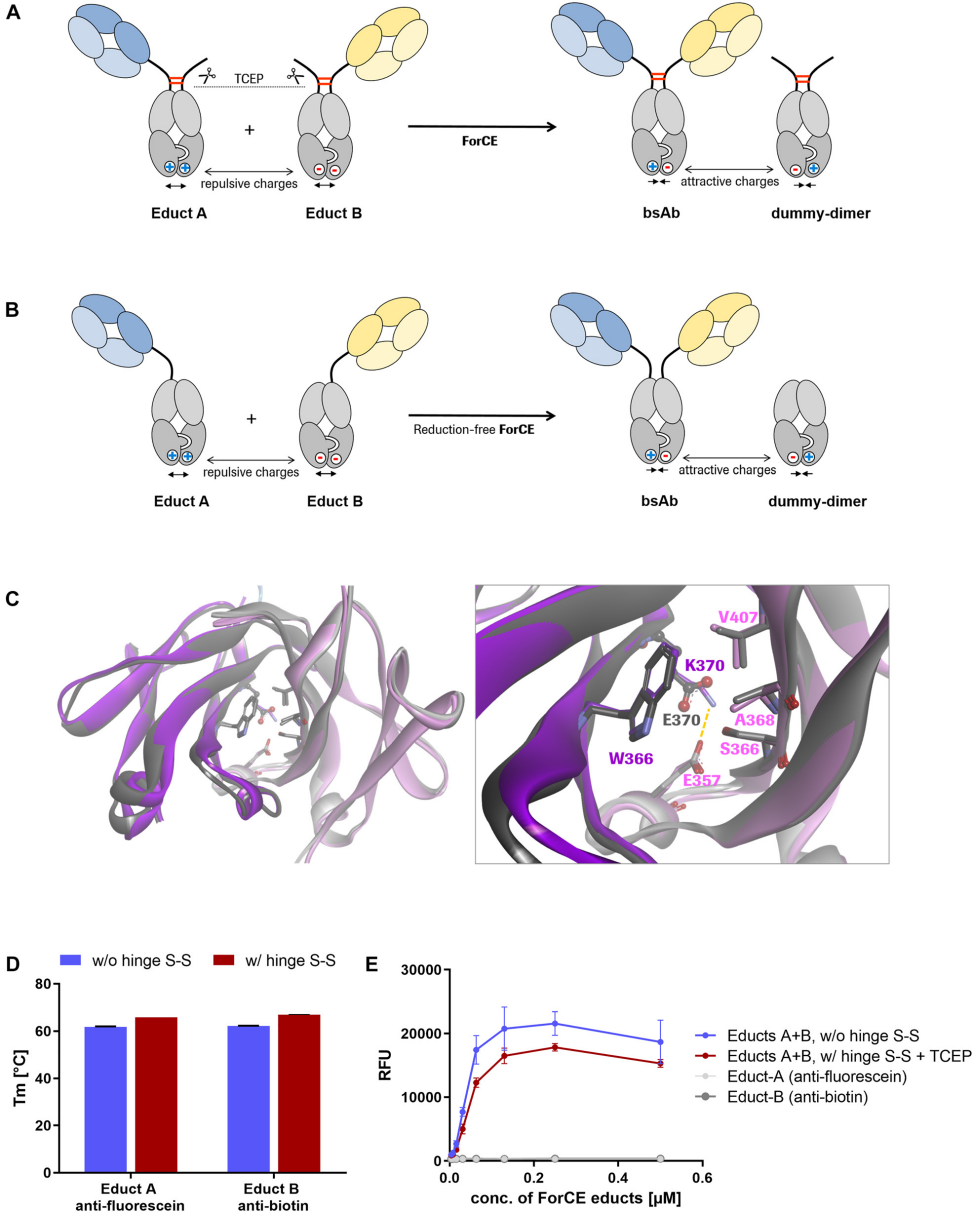
CH3-driven chain exchange enabled antibodies with and without interchain hinge-disulfides. (A) The Format Chain Exchange (ForCE) reaction. Two monospecific educts undergo chain exchange into bispecific antibodies under mild reducing conditions (in the presence of tris(2-carboxyethyl)phosphine (TCEP)). Educt A carries repulsive positive charges in the interface between the heavy chain and dummy chain, Educt B has two opposing negative charges. In the product, the attractive charges facilitate the stable formation of the bsAb, in which the hinge disulfide bridges re-oxidize (TCEP degrades over time). Fc portions (CH2/CH3) are depicted in gray. (B) The reduction-free ForCE reaction. The two monospecific educts undergo charge-mediated heavy chain recombination at physiological conditions and assemble into a bispecific antibody and a dummy-dimer molecule. (C) The CH3 domains of the crystal structure 4NQS representing the regular KiH (magenta: Knob [W366]; pink: Hole [S366, A368 and V407]) is superimposed with the single mutant of the Knob side (dark gray) and the Hole side (light gray) as present in educt B. In the latter, the gray ribbons are slightly more distant than seen in the reference KiH structure, reflecting the slightly destabilized nature of the prodrug (left panel). A zoom into the specific interaction of the KiH amino acids as well as on the single mutation K370E, that leads to a disruption of the salt bridge (yellow dotted line) with E357 in educt B (right panel). (D) Thermal stability of educt molecules with (traditional ForCE) and without disulfide-bridged hinge regions (ForCE under physiological conditions) was compared by differential scanning fluorimetry in three independent experiments, in triplicates. Depicted are mean melting temperatures and SD melting temperatures were greater than 60 °C, revealing an acceptable molecule stability. Educts contained Fabs that bind to conjugated fluorescein <Fluo> or conjugated biotin <Bio> (Dengl et al. 2015; Dengl et al. 2020). (E) A sandwich ELISA assay was performed to show the formation of the bsAb. BSA-Fluorescein was complexed on a plate. Educts A and B, with or without hinge disulfides (S–S), were mixed at 5 µM for 1 h at 37 °C and added to the plates. After several washing steps, biotinylated-Cy5 was added and unbound dye removed. The bsAb-mediated capture of Bio-Cy5 was detected in a microplate reader (ex 649 nm, em 670 nm). Results are expressed as mean and SD from triplicate wells. Representative plot of three independent experiments is shown.

In this previous report, we showed that fully oxidized, disulfide-stabilized bsAbs can be generated from large combinatorial matrices. ForCE generates bispecific knob-into-hole antibodies by combining complementary educts that harbor dummies containing knob or hole CH3 domains along with mutations that partially destabilize the CH3–CH3 interface (Figure 1A). The ForCE reaction is initiated by limited reduction of the hinge-disulfides of educts, after which the heavy chains spontaneously exchange. This converts the partially destabilized CH3-dummy dimers into stable CH3–CH3 dimer products. As a by-product, stable dummy-dummy dimers are also formed. Because the hinge regions of the educts connect the productive sides to the dummies through disulfides, initial reduction to start the reaction is essential, and product re-oxidation is also required (Dengl et al. 2020). Figure 1B shows our adaptation to enable chain exchange not only in controlled conditions in test tubes (including chemical reduction), but also under physiological conditions. We examined whether the mutation of cysteine residues in the hinge region that naturally form interchain disulfide bridges could abrogate the necessity of reducing agents to start the reaction. We replaced cysteine residues with serine. Hence, the only driving forces of this chain exchange are engineered charge repulsions placed into the CH3 domains of the precursors. Regular human CH3 dimer interfaces contain two apposed charged residues, E357 and K370, constituting an attractive electrostatic interaction. In the precursors, attraction at this position is converted to repulsion by charge inversion in the dummy chain CH3 domains (E357K or K370E). This leads to partially destabilized interfaces of heavy and dummy chains. Despite carrying partially flawed interfaces, and despite having the hinge-disulfides removed, dummy-containing precursors can be produced as defined entities of correct composition, as shown by analytical size exclusion chromatography, and confirmed by non-reducing and reducing SDS-PAGE analyses (Figure S1). These analyses confirm that the interchain disulfides in the hinge regions are not essential for proper generation and stability of knob-into-hole heavy chain heterodimers, even in the presence of partially repulsive interface mutations. Such molecules are of sufficient stability to be expressed at high levels and possess favorable biophysical properties, similar to standard IgGs (see below). The structural composition of the precursor CH3 domains in comparison to the regular KiH-CH3 is shown in Figure 1C. Upon completion of the chain exchange reaction, both the bsAb product and the dummy-dimer by-product regain an attractive charge-pair in their CH3 interfaces. As a result, the products and by-products are thermodynamically favored over the precursor molecules. This – in turn – drives the exchange reaction.

To evaluate the influence of the elimination of interchain hinge disulfides on protein stability, we compared the physical stability of hinge containing versus non-disulfide bridged precursor molecules towards thermal stress. The disulfide-stabilized molecules were slightly more thermostable; however, both species maintained their molecular integrity to temperatures greater than 60 °C (Figure 1D). Plate-based bridging assays revealed that the ForCE reaction under reducing (for hinge-containing educts) and non-reducing (for non-disulfide bridged educts) conditions is equally efficient and yields functional bispecific antibodies (Figure 1E).

This demonstrates that exchange reactions driven by complementary partially destabilized CH3 interfaces can be achieved with entities that do not harbor hinge interchain disulfides. The fact that exchange of those molecules does not require initiation by reduction indicates that spontaneous exchange should also function under physiological conditions.

### The Prodrug-Activating Chain Exchange (PACE) concept

Exchange reactions that occur spontaneously, without reduction, and upon the encounter of two complementary educts provide the opportunity to function not only under controlled conditions *in vitro* but also under physiological conditions. The driving force of these exchange reactions are partially destabilizing CH3 interface mutations, which resolve into stable interfaces in the reaction products (Figure 1A [Dengl et al. 2020]). Since the reactant molecules do not carry any further modifications compared to wild type domain sequences, this chain exchange principle should also be applicable to other antibody derivatives that harbor such CH3 domain dimers, provided they encounter each other in close proximity (i.e., at sufficient local concentrations).

**Figure 2:**
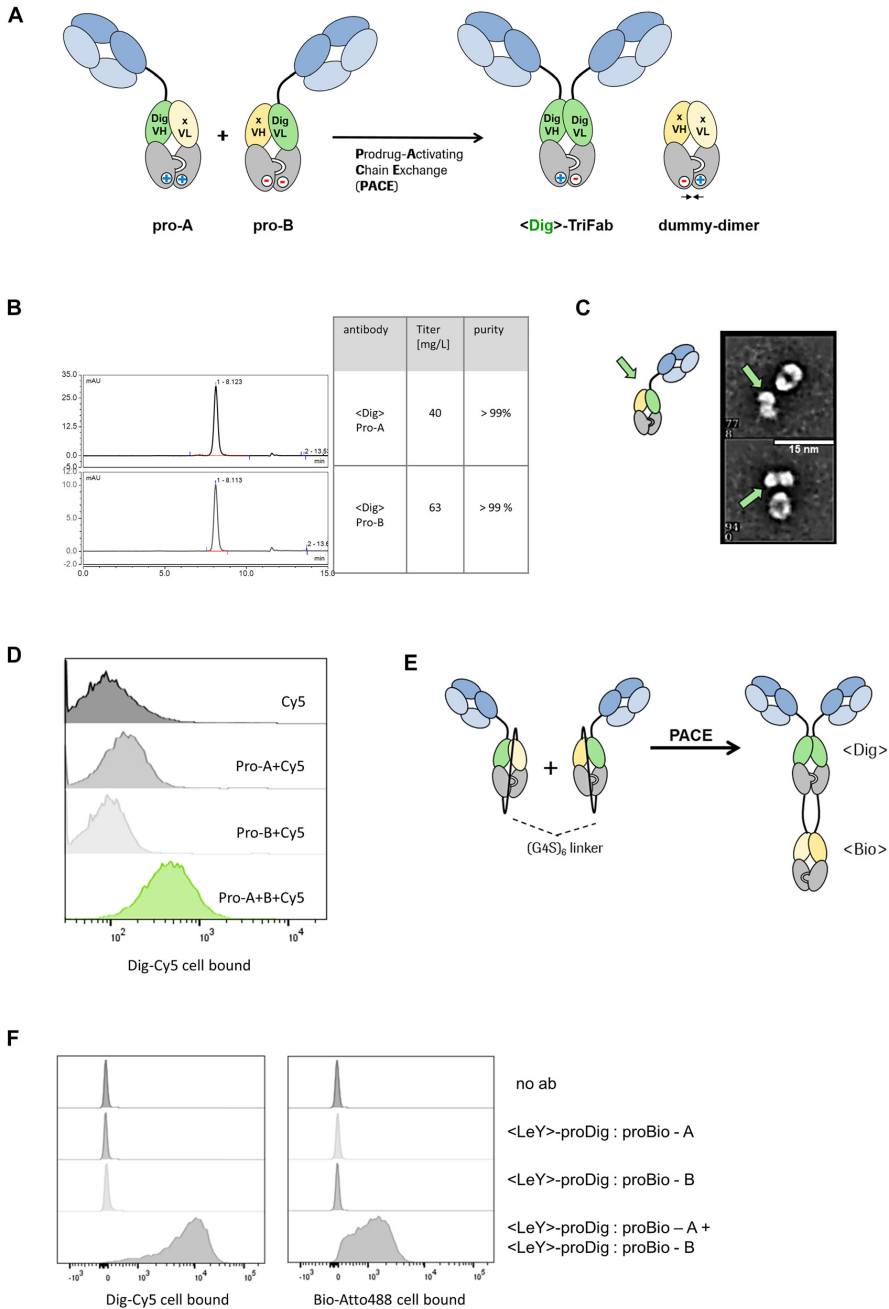
Prodrug-Activating Chain Exchange (PACE). (A) PACE. Two inactive prodrug molecules (pro-A and pro-B) undergo charge-mediated heavy chain recombination and assemble into a productive digoxigenin binder and a dummy-dimer molecule. (B) Precursor molecules can be produced at high yields (>40 mg/l expression volume) and high purity (>99%), indicated by the analytical SEC chromatogram. (C) Transmission electron microscopy images reveal one-armed molecules of expected size/shape. For details, see also (Dickopf et al. 2019). (D) The combination of pro-A and pro-B in presence of digoxigeninylated Cy5 dye displays the formation of an active Dig binder and the complexation of the dye on the cell surface of MCF-7 cells. (E) Monospecific precursors convert into a trispecific product molecule. Both precursors harbor a dummy chain fused to the C terminal end of the heavy chain. Instead of an irrelevant Fv in the dummy, a VH/VL that originates from a biotin binder was integrated respectively. Upon chain exchange, both the digoxigenin and the biotin binder are formed. (F) Cytometric analysis indicates the complexation of both biotin-Atto488 and dig-Cy5 on the cell surface of LeY(+)-MCF-7 cells and demonstrates the formation of trispecific binders.

TriFabs are a type of CH3-dimer containing, IgG-derived bispecific antibody format. They consist of two regular Fab arms for tumor targeting and a central third binding entity that substitutes the regular CH2 domains. TriFabs can bind antigens such as haptens (e.g., digoxigenin) or proteins such as CD3 with their central binding site (Dickopf et al. 2019; Mayer et al. 2015). TriFabs do not contain interchain disulfides in their hinge-like linkers that connect their two Fab arms to one Fv-(CH3)2 entity. The molecular composition of TriFabs is hence very similar to IgGs without hinge interchain disulfides, with the sole exception that the CH2 domain is replaced by an Fv (Figure 2A, right).

An interesting feature of TriFabs is that a vertical separation along the heavy chain interface splits the VH and VL of the Dig binder into two inactive halves. We therefore used this format to test whether the ForCE-like chain exchange can be applied to generate prodrugs, and to convert those to active products by chain exchange (Figure 2A).

To demonstrate the applicability of this approach, proof-of-concept experiments were performed with TriFabs that harbored Digoxigenin (Dig) binding prodrug entities (as the generation of targeted Dig-binders can easily be monitored by robust assays [Metz et al. 2011]). Hence, a PACE reaction generates an active <Dig>-TriFab from two inactive prodrug molecules, pro-A and pro-B (Figure 2A). Each of these harbor one functional cell surface binding Fab, and another Fab-like entity composed of a VH-VL heterodimer fused to knob-into-hole CH3 domains. The Fab-like entities harbor the prodrug functionalities. They are composed of either VH-CH3 of a Dig binder paired with VL-CH3 of an irrelevant antibody (pro-A), or VL-CH3 of a Dig binder paired with VH-CH3 of an irrelevant antibody (pro-B), respectively. The VH- or VL-CH3 fusions that do not recognize Dig and contain no additional cell surface targeting entities are termed ‘dummy chains’. Thus, VH and VL of the Dig binder are placed on two separate half-TriFabs, each of which equipped with a dummy chain consisting of a non-targeting VH or VL and a CH3 domain. The dummy chains provide stability, enable efficient protein production, and prevent the exposure of free hydrophobic VH or VL domain interfaces. The PACE reaction that converts the pro-Dig binder to an active moiety is defined by a molecular exchange of the heavy- and dummy-chains of two complementary prodrugs, similar to what has been demonstrated in ForCE (Dengl et al. 2020). This yields a TriFab as a functional Digoxigenin binder and a dummy heterodimer as a by-product.

Figure 2B shows that educt molecules of expected size can be produced with high yield by transient expression (>40 mg/L) and with high purity (>98%). Transmission electron microscopy of the non-disulfide stabilized molecules shows that the educts have shapes that are in line with those expected for one-armed, properly assembled entities (Figure 2C). Proof of function was determined by combining two prodrugs in the presence of a Cy5-labeled digoxigenin compound on MCF-7 target cells that express the LeY antigen, which is recognized by the Fab arms of the TriFab. FACS analyses reveal that PACE generates LeY targeting bsAbs that harbor functional Dig-binding entities and retain the dye on the cell surface (Figure 2D).

We further tested whether other formats are suitable to undergo the PACE reaction. Therefore we covalently attached the dummy chain with a 6×(G4S) linker to the C-terminal end of the heavy chain. Additionally, instead of the irrelevant dummy CDRs, we used variable fragments (VH, VL) of a biotin binder. The resulting product should contain two new functional binders, a digoxigenin binder and a biotin binder (Figure 2E). A cytometry-based readout with Dig-Cy5 and Bio-Atto488 confirmed that the two new binding sites are indeed functional after chain exchange (Figure 2F), and become targeted to cell surfaces by the Fab arms of the TriFab derivative.

### PACE generates an active TriFab T cell engager from two inactive prodrugs

By crosslinking the T cell receptor on T lymphocytes and tumor cells, TCBs induce potent T cell-mediated cytotoxicity at the tumor site (Labrijn et al. 2019).

In an earlier report we could show that the TriFab-TCB format has a high molecular flexibility and induces efficient T cell-dependent immune responses on specific tumor cells (Dickopf et al. 2019). Due to its symmetric composition, we wondered whether it is possible to use the PACE technology to generate active TCBs from two inactive precursors, similar to what we showed with Dig- and Bio-binders.

**Figure 3:**
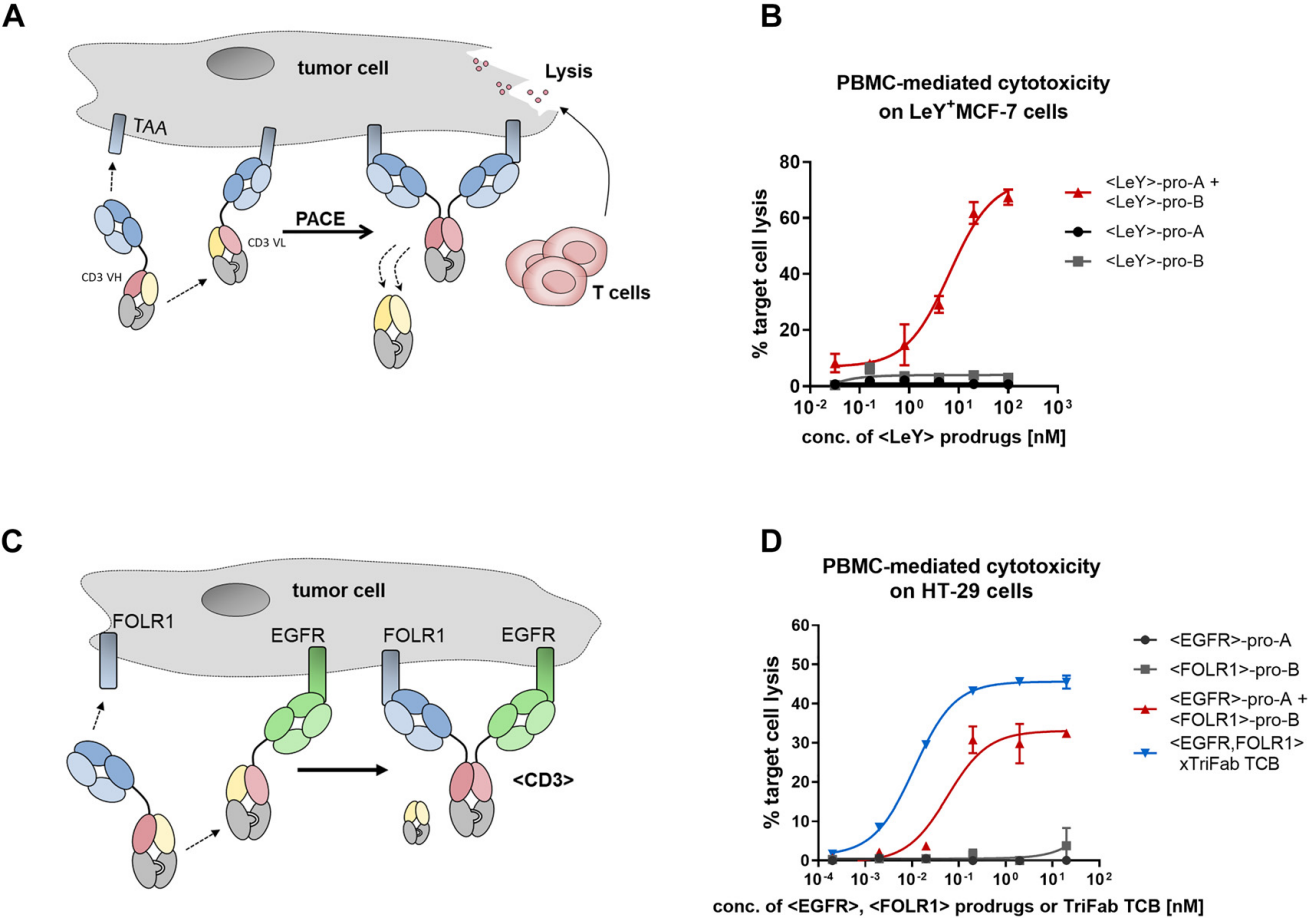
Prodrug activation by chain exchange and *in vitro* activity towards target cells. (A) Accumulation on the cell surface as result of tumor-associated antigens (TAA) binding (blue domains) leads to a high local concentration of the prodrugs and induces the thermodynamically driven reaction towards the product molecule – an active T cell engager that induces tumor cell lysis. (B) Initial proof-of-concept experiment. Pro-A and pro-B were designed with cell surface binders that recognize the tumor-associated carbohydrate antigen Lewis Y (LeY, Brinkmann et al. 1991). To assess Prodrug-Activating Chain Exchange (PACE)-induced activation of lymphocytes, peripheral blood mononuclear cell (PBMC) were isolated from the blood of healthy human donors and co-cultivated with LeY^+^-MCF-7 cells for 48 h with antibodies and target cells. Percentage of target cell killing (which depends on binding of at least one arm to the tumor cell surface and of the CD3-binder to T-cells) was measured by lactate dehydrogenase (LDH) release of dead cells. Results are expressed as mean and SD from triplicate wells and fitted with a three-parameter non-linear regression. Representative killing curve of four independent experiments (including different donors) is shown. (C) The dual-targeted PACE reaction. The two prodrugs are designed to target two distinct TAA: epidermal growth factor receptor (EGFR) and FOLR1. Through PACE, trispecific active TCBs are generated. (D) PBMC killing with PACE molecules on HT-29 cells, as illustrated in panel (C). Results are expressed as mean and SD from triplicate wells and fitted with a three-parameter non-linear regression fit using GraphPad Prism software. Representative killing curve of three independent experiments (including different donors) is shown.

We designed PACE reactants that generate an active TriFab T cell engager from two inactive prodrug molecules (Figure 3A). To establish the feasibility of PACE, we first used prodrug molecules that target the carbohydrate antigen LeY, which is highly expressed on MCF-7 cells (Brinkmann et al. 1991). This target was chosen to facilitate a high local accumulation of prodrug molecules on the target cell. MCF-7 cells were co-incubated with human PBMCs and <LeY> prodrug molecules. While there was no killing detectable with the individual prodrugs, the simultaneous application of both prodrugs led to a dose-dependent elimination of tumor cells (Figure 3B). Similar results were obtained in a bispecific approach where FOLR1 and EGFR were targeted on HT-29 cells (Figure 3C, D).

### PACE generates active TCBs *in vivo* and confers growth inhibition of tumor xenografts in mice

**Figure 4:**
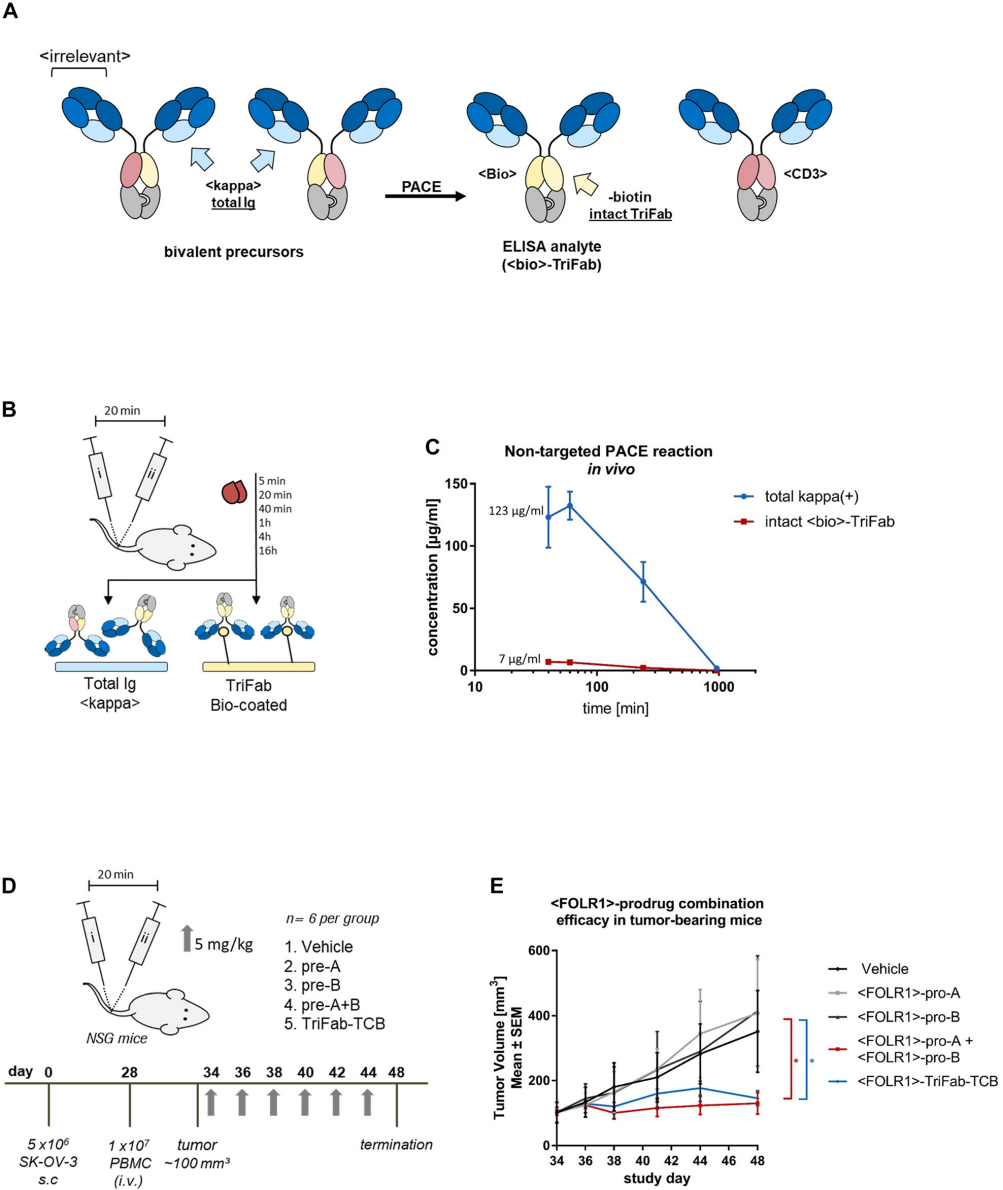
Determining *in vivo* characteristics of Prodrug-Activating Chain Exchange (PACE) molecules. (A) In order to monitor in-serum shuffling and TriFab (PACE product) stability in mice, bivalent <LeY> prodrugs were generated. The CD3-specific variable fragment is paired with a VH or VL of a biotin binder respectively. Upon chain exchange, functional LeY-binding <CD3> and <Bio> TriFabs are generated. (B) NSG mice (*n* = 3 per group) were administered with an i.v. injection of prodrug molecules alone or in combination (5 mg/kg, with a 20 min time span in-between). Serum samples were collected at multiple time points post administration. Total antibody-like content (kappa(+)) was measured by applying serum samples to an anti-kappa light chain ELISA. <Bio>-product molecules that occur by an unspecific in-serum shuffling can be captured to the ELISA plate by immobilized biotin. (C) Absorbance values of each serum sample were determined in triplicates. A pre-assembled TriFab-control was used to generate a standard curve. Results are expressed as mean and SD from triplicates. (D) TriFab-PACE *in vivo* efficacy was evaluated in a SK-OV-3 ovarian cancer model. NSG mice were inoculated with FOLR1-expressing SK-OV-3 cells. On day 28 after cell inoculation, fresh human PBMCs were injected i.v. Six days after PBMC engraftment (day 34) mice were randomized into five groups. Prodrugs were injected i.v. on every second day (pro-A, pro-B, pre-A + B; TriFab TCB control, 5 mg/kg) and compared to a vehicle group (buffer control). The combined group (pro-A + pro-B) group was injected sequentially with a 20 min time interval between injections to avoid the serum peak concentration. (E) Tumor growth was monitored and volume was calculated as follows: (length × (width)^2^)/2. GraphPad Prism software version 6 was used for statistical analysis. Statistical comparisons between vehicle and treatment groups were performed using one-way analysis of variance (ANOVA) and Tukey’s multiple comparison test. *p* values less than 0.05 were considered statistically significant (*p ≤ 0.05).

A key requirement for PACE to provide a safety advantage over an active TCB is that the exchange reactions happen preferentially upon co-accumulation on target sites (cell surfaces, tissues) but not at significant levels in the circulation. To evaluate whether in-serum assembly independent of accumulation on the cell surface represents a major issue, we applied prodrug molecules that target LeY in a bivalent manner and, upon chain exchange, generate a biotin-binding site as a by-product (Figure 4A). The generation of the biotin-binding site facilitates the detection of productive chain exchange in a sandwich ELISA as shown in Figure 4B. To assess potential premature activation of these constructs, we intravenously injected 5 mg/kg of each prodrug into tumor-free mice (which do not express LeY). Pro-A and pro-B were applied with a 20 min time difference to allow initial distribution of the first prodrug before injecting the second prodrug. We then took blood samples at various time points and analyzed the serum for assembled biotin-binding TriFabs. Detection of such molecules would result from premature exchange in solution, since mice do not express LeY target antigens that could trigger on-cell co-accumulation. Figure 4C reveals that some degree of in-solution chain exchange does indeed occur, yet at a low percentage level. The levels of in-solution exchanged products are approximately 6% at early time points (at peak concentrations), which do not increase over time. They rather decrease to below detection levels within 5 h after application, probably due to their PK properties as FcRn-binding deficient TriFabs and rapid clearance. Since there is no functional Fc region present in the TriFab-TCB format, the molecules are more rapidly cleared than IgGs with FcRn-binding Fc regions (Schlothauer et al. 2013). However, due to their size of >50 kDa, they may be retained longer as compared to BiTE-like molecules, which undergo renal clearance (Yuraszeck et al. 2017).

Building on the findings described above, we analyzed the *in vivo* efficacy of human FOLR1-targeting PACE prodrug combinations (which does not bind murine FOLR1) in comparison to vehicle and pre-assembled active TriFab-TCB controls. Therefore, subcutaneous FOLR1 expressing SK-OV-3 xenografts were established in NSG mice and treated upon reaching a size of ∼100 mm^3^. PBMCs from healthy human donors were injected six days prior to the first dosing. Based on the serum half-life of the PACE molecules, we administered each of the antibody derivatives at concentrations of 5 mg/kg every second day to ensure a sufficient serum concentration (Figure 4D). After two weeks with six treatments in total, both the prodrug combination and the TriFab-TCB showed a significant reduction in tumor volumes compared to all other controls (Figure 4E). This in turn shows that the *in vivo*-generated bispecific T cell engagers induce an efficient anti-tumor response in human xenografts. The relative contributions of on-cell assembly and cell binding of in-solution assembled TriFab molecules (or a hybrid of both mechanisms) cannot be fully distinguished in this setting.

## Discussion

Cytolytic activities of TCBs have proven to be effective to fight tumors (Trabolsi et al. 2019). Yet, only few TAA are currently amenable as targets for TCB therapies. The reason is their presence (albeit to much lower degree) in certain healthy tissues, particularly of epithelial origin (Ellerman 2019). Thus, technologies that enable a safe target space expansion towards TAAs are highly desired. This challenge may be addressed by specific activation of prodrug TCBs at the tumor site. The focus in the field of conditional immunotherapy is currently predominantly on proteolytic cleavage of covalently attached anti-CD3-masking caps (or domains) in the tumor microenvironment (Boustany et al. 2018; Jack Lin 2018; Minogue et al. 2019; Trang et al. 2019). Additional early pre-clinical concepts address the possibility to reconstitute a functional CD3-Fv from VH or VL-only hemibody prodrugs on the surfaces of target cells. This is based on the tumor-specific expression of two different antigens that are not found co-expressed in normal tissues, or applying a combination of both the protease-activation and split Fv assembly (Minogue et al. 2019).

The herein described PACE-mediated prodrug activation differs from the above-mentioned approaches in several aspects. It neither depends on environmental triggers (such as proteases) for activation, nor harbors incomplete antibody fragments with unpaired domains. Because the reconstitution of prodrug-binders does not depend on proteolytic cleavage, PACE does not face restrictions of protease-dependent approaches such as mandatory and highly specific expression of a protease in the tumor microenvironment, or undesired activation by non-tumor proteases.

TriFab-PACE prodrugs are composed of fully assembled but nonfunctional antibody modules. Therefore, they are stable, with favorable IgG-like expression levels and biophysical properties. The lack of unpaired domains (domains with free interfaces, VH or VL only), which are frequently associated with elevated aggregation propensities and stability/developability problems, provides favorable biophysical properties. Because of their composition with fully assembled antibody domains, PACE prodrugs also follow an alternative mode of activation. While split Fvs require simple VH-VL association to regain activity, PACE depends on a multistep domain/chain exchange reaction (as opposed to an association reaction). This exchange reaction also converts monovalent target cell binders to active molecules that are retained on target cells in a bivalent manner with avidity-increased binding strength. A unique feature is also the driving force for the activating exchange reaction, provided by engineered CH3 interfaces. For activation of PACE prodrugs on target cells, both educts must encounter each other in sufficient concentrations to trigger effective chain-exchange reactions. Specific tumor accumulation of prodrugs requires binding of their targeting arms to cognate cell surface antigens. The degree of prodrug accumulation is hence dependent on the density of the targeted antigens. Tumor cell surface targets that enable effective PACE reactions are therefore expected to be those that are highly expressed. This restriction, however, may also be advantageous as many tumor-associated target antigens are not expressed with ‘absolute tumor specificity’ but are instead expressed to a lower level also on certain normal, healthy cells. Thus, a requirement of high antigen density (threshold concentration) for prodrug activation may prevent non-desired activity towards low antigen expressing normal/healthy cells.

After encountering each other in sufficient concentrations, the prodrugs must release (i.e., dissociate) their dummy chains simultaneously in close proximity to each other, and undergo a crosswise chain exchange. The exchange reaction is favored because the products possess fully complementary CH3 interfaces while educts harbor partially destabilized interfaces. This resolution of partially repulsive interfaces into completely complementary (i.e., attractive) interfaces results in a directed, close to irreversible reaction once products have been formed – a feature that might differ from mere VH-VL association – based hemibody approaches (Banaszek et al. 2019). One additional particular feature is the formation of dummy dimers as by-products of the exchange reaction. The dummy dimers also contain re-constituted VH-VL combinations, providing the option to generate additional binding functionalities that may further increase the efficacy of the activated TCBs (Kobold et al. 2018; Krupka et al. 2016).

The chain exchange reaction as the underlying principle of PACE requires two complementary prodrugs to be in close proximity. Our previously established ForCE platform has the same requirement but aims at generating bsAb binder-format matrices (Figure 1B) under controlled test-tube conditions in solution. For PACE, such efficient exchange in solution is not desired, as it might cause premature prodrug conversion. Our *in vivo* analysis revealed indeed some (6%) in-solution exchange that was observed at peak concentrations and early time-points after prodrug application. Active bsAbs could hence be present in the circulation at those time points.

Systemic administration of a monospecific FOLR1-targeted prodrug combination led to a significant growth inhibition of established SK-OV-3 tumor xenografts in mice. Tumor regression was observed to the same extent as with the pre-formed active control TCB under conditions that cause very little premature in-solution activation. This shows that active TCBs are generated from systemically applied prodrugs and are bound to tumor cells, where they recruit T-cells. We cannot exclude, however that this efficacy may not only reflect on-cell activation, but also reflects contributions by the small fraction of in-serum exchanged TCBs accumulating in the tumor from the circulation.

Prodrug applications that result in significant undesired premature prodrug activation in the circulation would not have a marked benefit over a pre-assembled TCB, as this might lead to similar toxicity issues. Options to address potential premature prodrug activation in the future, and to further progress chain-exchange-based technologies may therefore include the modulation of chain exchange reactants or of application schemes, such as extending the time between 1st and 2nd prodrug application (a pre-targeted approach) for PACE derivatives that by themselves have a limited serum half-life.

Finally, chain-exchange-based PACE technologies are not limited to the conversion of monospecific targeted prodrugs into bispecific antibodies. A further specificity level can be added by expanding monospecific PACE to dual target-specificity, i.e., targeting two different cell surface antigens on the same cell (Figure 3C). Prodrug activation thereby generates a trispecific molecule (or for sc-linker formats shown in Figure 2E even tetraspecific entities).

## Materials and methods

### Design, production and biochemical characterization of antibody derivatives

For antibody generation we used the following target antigen binders: <LeY> (clone mAb B3 [Brinkmann et al. 1991]), <FOLR1> (clone 16D5 [Geiger et al. 2020]), <EGFR> (clone C225 [Huang et al. 1999]), Nadaceptin (Dickopf et al. 2019), <CD3> (40G5c [Chen et al. 2015]). Knob-into-hole mutations (knob: T366W; hole: T366S, L368A, Y407V) were introduced into CH3 domains (Ridgway et al. 1996). All constructs were produced via transient transfection with the Expi293 Expression System (Thermo Fisher) according to the manufacturer’s recommendations. The transcription was driven by a CMV promoter. As a signal peptide an IgG leader sequence of Ig heavy chain V region was used (Zuo et al. 2000). Supernatants were harvested six days following transfection, spun down at 4000 g and sterile filtered through a 0.22 μm filter unit (Thermo Fisher Scientific, Waltham, MA, USA). For affinity chromatography supernatants were applied to 2  ×  5 ml HiTrapKappaSelect or HiTrapLambdaFabSelect (GE, 17-5458-11, 17-5482-11, Boston, MA, USA) and processed according to the manufacturer’s recommendations. Bound proteins were eluted with 50 mM sodium citrate (KappaSelect) or 0.1 M sodium acetate (LambdaSelect) buffer at pH 2.7. As a neutralization buffer, 1 M TRIS (pH 9) was added to the fractions (*v* = 5% of fractionation volume). Samples were then applied to a HiLoad^®^ 26/600 Superdex^®^ 200 pg (GE, 28989336, Boston, MA, USA) and processed as recommended by the manufacturer. As a running buffer a 20 mM histidine, 140 mM sodium chloride solution at pH 6.0 was used. Analytical SEC was performed using the HPLC device Dionex UltiMate 3000 (Thermo Fisher) with a BioSuite Diol (OH) Column (Waters, cat#186002165) (Flow: 0.5 ml/min, RT).

### Differential scanning fluorimetry (DSF)

Differential scanning fluorimetry (DSF) was performed in triplicates in a 384-well format using the LightCycler instrument (Roche, 05015243001).

SYPRO orange (Sigma, S5692) was first diluted 100-fold in water and another 10-fold in PBS. Antibodies were diluted in PBS to a final concentration of 1 µM.

One microliter of the SYPRO orange solution was then combined with 4 µl sample solution in a 384-well plate. Fluorescence (465–580 nm) was measured with increasing temperature (gradient 0.06 °C/s, start: 20 °C; end: 95 °C). The melting point was defined as d([RFU]/dT) and calculated with the LightCycler Software.

### Negative stain transmission electron microscopy (NS-TEM)

For grid preparation, samples were diluted (concentration = ∼5 μg/ml) in D-phosphate buffered saline. Four microliters of the dilution were adsorbed to glow-discharged 400 mesh carbon coated Parlodion copper grids, washed with three drops of water, incubated with 3 μl of a solution containing tobacco mosaic virus, further washed with two drops of water and finally stained with two drops of 2% uranyl acetate. Samples were then imaged using a Tecnai12 transmission electron microscope (FEI, Eindhoven, The Netherlands) operating at 120 kV. Electron micrographs were recorded on a 2048 by 2048 pixel charged-coupled device camera (Gloor Instruments, Kloten, Switzerland) at a nominal magnification of ×195,000 yielding a final pixel size of 0.296 nm on the specimen level. Alternatively, samples were imaged using a FEI Tecnai G2 Spirit TEM (FEI, Eindhoven, The Netherlands) operating at 80 kV. Electron micrographs were then recorded on a 2048 by 2048 pixel charge-coupled device camera (Veleta Soft Imaging Systems, EMSIS, Münster, Germany) at a nominal magnification of ×135,000 yielding a final pixel size of 0.33 nm on the specimen level. Images were processed by reference-free alignment on manually selected particles from recorded images using the EMAN2 image processing package. The extracted particles were aligned and classified by multivariate statistical analysis yielding two-dimensional (2D) class averages. Additionally, when class averaging was not possible, raw images of particles were also manually stained for clarity using Photoshop (Adobe Systems, San José, CA, USA).

### Cell culture and reagents

MCF-7 (ATCC^®^ HTB-22™), SK-OV-3 (ATCC^®^ HTB-77, Manassas, VI, USA), HT-29 (ATCC^®^ HTB-38™), were cultivated in Roswell Park Memorial Institute (RPMI) Medium 1640 (Gibco 31870-025, Waltham, MA, USA) substituted with 10% fetal calf serum (FCS) (Biowest, S181B) and 2 mm l-glutamine (Gibco 25030-81, Waltham, MA, USA). For detaching cells, Accutase^®^ solution (Sigma, A6964) was used. Cells were counted using a Vi-CELL XR Analyzer (Beckman Coulter, Brea, CA, USA).

### Flow cytometry

Binding to target cells was confirmed via flow cytometry. A total of 3 × 10^5^ cells were incubated with 100 nM of antibody derivatives in FACS buffer (PBS [PAN Biotech, P04-36500] with 2% FCS) for 1 h at 37 °C. Then, cells were washed twice with PBS and incubated for an additional hour with hapten-conjugated dye (Dig-Cy5, Bio-atto488) in FACS buffer on ice. Following two washes with PBS, the cells were analyzed for dye intensity using a FACS CantoII instrument (BD biosciences). FlowJo (BD) software was used for data analysis and visualization.

### PBMC-mediated cytotoxicity assays

About 2.0 × 10^4^ cells were seeded out in standard RPMI1640 (10% FCS, 2 mM glutamine) the day before PBMC co-cultivation in 96-well plates (day 0). On day 1, fresh whole blood from healthy human donors was processed according to the manufacturer’s recommendations using Ficoll^®^ Paque Plus (GE Healthcare, Chicago, IL, USA) and Leucosep™ centrifuge tubes (Greiner Bio-one, Kremsmünster, Austria). Mononuclear cells were stained for viability with trypan blue and counted using a conventional Neubauer chamber. As assay media for co-culturing RPMI1640 (1% FCS, 2 mm glutamine) was used. A dilution series of respective antibodies was prepared in assay media and added to target cells. About 2.0 × 10^5^ PBMC were added to each well resulting in a total volume of 200 μl. Lactate dehydrogenase (LDH) release was measured after 48 h using the Cytotoxicity-Detection Kit (from Sigma, by Roche 11644793001, Basel, Switzerland) according to the manufacturer’s recommendations (including calculations and high control using 2% Triton-X100). The means and standard deviations (SD) from triplicate wells were plotted and fitted with a 3-parameter non-linear regression using GraphPad Prism 7 software (GraphPad Software, San Diego, CA, USA).

### *In vivo* studies and analytics

#### Determining pre-mature shuffling

All animal studies were approved by the local government and mice were handled according to committed guidelines of the Federation for Laboratory Animal Science Associations. The animal facility is accredited by the Association for Assessment and Accreditation of Laboratory Animal Care. Single dose pharmacokinetics studies were performed in female 6–8 week old NSG (NOD.Cg-Prkdcscid Il2rgtm1Wjl/SzJ, Charles River Laboratories, Lyon, France) mice. Mice were administered with an intravenous (i.v.) injection of prodrug molecules (TriFab-TCB control, prodrug A, prodrug B; 5 mg/kg; *n* = 3). Serum was collected at multiple time points post administration (5 min, 20 min, 40 min, 1 h, 4 h, 16 h). The combination treatment group was injected with pro-A followed by administration of PACE pro-B after 20 min and blood sampling was performed 40 min, 1 h, 4 h, 16 h after first dose. Serum samples were subsequently analyzed for compound presence in a four-step solid phase ELISA. All antibody dilutions were prepared in low cross dilution buffer (Candor Biosciences; Wangen, Germany) and subsequent dilutions were performed in low cross buffer with 2% pooled mouse serum. All reagents or samples were incubated in 100 µl per well at room temperature on a shaker at 400 rpm. The washing buffer was applied in 300 µl volumes. Each washing step included three cycles. For both assays, the TriFab-control was used as a standard to generate a standard curve in the range of 0.15–10 ng/mL.

Total antibody-like protein (kappa(+)) was detected by capturing with a biotinylated anti-human kappa light chain Fab (Clone M-1.7.10), coated onto a Streptavidin microtiter plate (SA-MTP, MicroCoat Biotechnologie GmbH, Bernried) and detection was performed with a digoxigenin-labeled anti human Ch1 antibody (Clone M-1.19.31) Biotin-binding antibodies (formed if two prodrugs assemble into a functional <Bio>-TriFab) were detected by capturing the antibody with biotinylated albumin, coated to a streptavidin microtiter plate. The albumin was biotinylated with a biotin to albumin ratio of 6:1, resulting in an albumin with more than one accessible biotin. The bound TriFab-analytes were detected by a digoxigenin-labeled anti human kappa light chain antibody at 0.5 μg/ml (Clone 1.7.10). In both assays, after washing, a polyclonal anti-Dig-HRP Fab (50 mU/ml) was added and ABTS (ThermoFisher) was used as a substrate. The signal of the colorigenic reaction is proportional to the concentration of the analyte and measured by an ELISA reader at 405 nm wavelength (reference wavelength: 490 nm). Absorbance values of each serum sample were determined in triplicates.

#### Determining the PACE efficacy *in vivo*

PACE *in vivo* efficacy was evaluated in a SK-OV-3 ovarian cancer model in female human peripheral blood mononuclear cell (PBMC) reconstituted NSG mice, aged 5–7 weeks. SK-OV-3 cells (5 × 10^6^ in 100 µl) were inoculated subcutaneously into the right flank. Animals were routinely checked for changes in body weight and general health. Tumor growth was monitored and volume was calculated as follows: (length × (width)^2^)/2. On day 28 after cell inoculation fresh human PBMCs (1 × 10^7^ in 100 µl PBS) were injected via the tail vein. For this purpose, human PBMCs from healthy volunteer donors were isolated using human lymphocyte separating tubes (human Pancoll, PAN-Biotech, Aidenbach, Germany) according to the manufacturer’s instructions. Six days after PBMC engraftment (day 34), mice were randomized into five groups (average tumor volume = 100 mm^3^; *n* = 6). Compounds were injected i.v. on every second day (pro-A, pro-B, pro-A + pro-B; TriFab TCB control, 5 mg/kg) and compared to a vehicle group (buffer control). The study had to be terminated on day 48 to prevent undesired graft-versus-host effects. The combination group (pro-A + pro-B) was injected in succession with a 20 min interval in-between to avoid the serum peak concentration. GraphPad Prism software version 6 (GraphPad Software Inc., San Diego, CA, USA) was used for statistical analysis. Statistical comparisons between vehicle and treatment groups were performed using one-way analysis of variance (ANOVA) and Tukey multiple comparison test. *p* values less than 0.05 were considered statistically significant (* = *p* ≤ 0.05).

## Supplementary Material

Supplementary Material
