# Supplementary material for: Prodrug-Activating Chain Exchange (PACE) converts targeted prodrug derivatives to functional bi- or multispecific antibodies
Source: Biol Chem. 2022 Jan 20;403(5-6):495–508. doi: 10.1515/hsz-2021-0401 (PMC9125802; doi:10.1515/hsz-2021-0401)
Supplement: Supplementary file 1 — Supplementary Material [file j_hsz-2021-0401_suppl.pdf]

Prodrug-Activating Chain Exchange (PACE) converts targeted prodrug derivatives to functional bi- or multispecific antibodies

Steffen Dickopf, Can Buldun, Vedran Vasic, Guy Georges, Carina Hage, Klaus Mayer, Matthias Forster, Uwe Wessels, Kay-Gunnar Stubenrauch, Jörg Benz, Andreas Ehler, Matthias E. Lauer, Philippe Ringler, Sebastian Kobold, Stefan Endres, Christian Klein and Ulrich Brinkmann

Supplementary material

Fig. S1

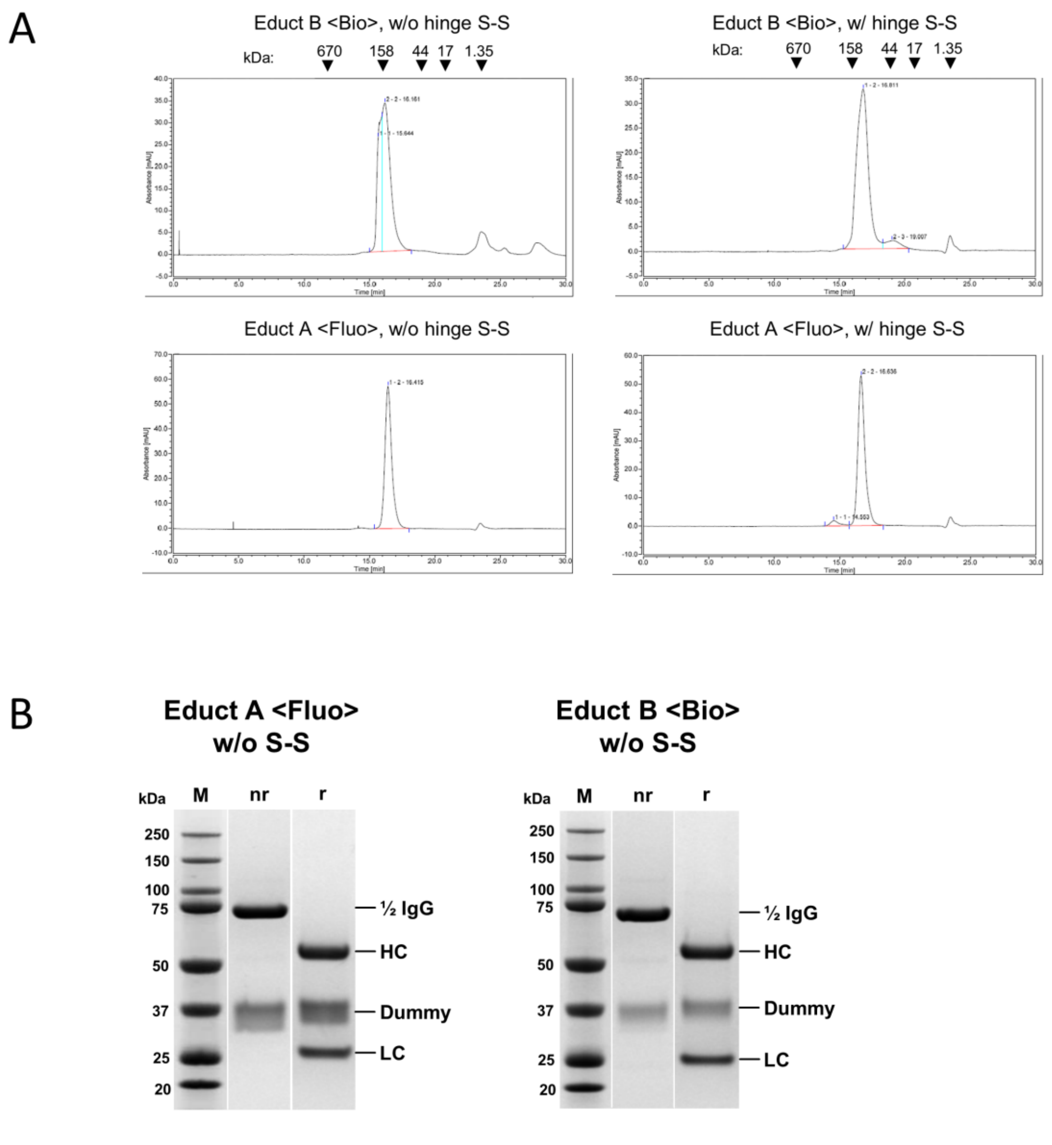

Figure S1 Analytics of ForCE educts from Figure 1.
(A) Analytical SEC chromatograms of ForCE educts with and without hinge disulfides. Fab arms are directed against biocytinamide or fluorescein: <Bio> and <Fluo>. Approximate molecular weights are indicated, as determined from a defined molecular weight standard. (B) SDS-PAGE and Coomassie blue staining of ForCE educts lacking hinge disulfides in non-reducing (nr) and

reducing conditions (r) indicates the assembly of heavy chain (HC), light chain (LC), and dummy chain in the expected stoichiometry.
